# Development of a groundwater quality index: GWQI, for the aquifers of the state of Bahia, Brazil using multivariable analyses

**DOI:** 10.1038/s41598-021-95912-9

**Published:** 2021-08-13

**Authors:** José Barbosa Filho, Iara Brandão de Oliveira

**Affiliations:** 1 Departamento de Ciências E Tecnologias Dos Materiais, Escola Politécnica, Universidade Federal da Bahia, Rua Aristides Novis, 2, Federação, Bahia, Salvador 40210-630 Brasil; 2 Departamento de Engenharia Ambiental, Escola Politécnica, Universidade Federal da Bahia, Rua Aristides Novis, 2, Federação, Bahia, Salvador 40210-630 Brasil

**Keywords:** Environmental sciences, Engineering

## Abstract

This work elaborated a groundwater quality index—GWQI, for the aquifers of the state of Bahia, Brazil, using multivariable analyses. Data from 600 wells located in the four hydrogeological domains: sedimentary, crystalline, karstic, and metasedimentary, were subjected to exploratory statistical analysis, and 22 out of 26 parameters were subjected to multivariable analysis using Statistica (Version 7.0). From the PCA, 5 factors were sufficient to participate in the index, due to sufficient explanation of the cumulative variance. The matrix of factorial loads (for 1–5 factors) indicated 9 parameters related to water quality and 4 hydrological, with factor loads above ± 0.50, to be part of the hierarchical cluster analysis. The dendrogram allowed to choose the 5 parameters related to groundwater quality, to participate in the GWQI (hardness, total residue, sulphate, fluoride and iron). From the multivariable analyses, three parameters from a previous index—NGWQI, were not selected for the GWQI: chloride (belongs to the hardness hierarchical group); pH (insignificant factor load); and nitrate (significant factor load only for 6 factors), also, not a regionalized variable. From the set of communality values (5 factors), the degree of relevance of each parameter was extracted. Based on these values, were determined the relative weights (w_i_) for the parameters. Using similar WQI-NSF formulation, a product of quality grades raised to a power, which is the weight of importance of each variable, the GWQI values were calculated. Spatialization of 1369 GWQI values, with the respective colors, on the map of the state of Bahia, revealed good correlation between the groundwater quality and the index quality classification. According to the literature on water quality indexing, the GWQI developed here, using emerging technologies, is a mathematical tool developed as specific index, as it was derived using limits for drinking water. This new index was tailored to represent the quality of the groundwater of the four hydrogeological domains of the state of Bahia. Although it has a regionalized application, its development, using, factor analysis, principal component analysis, and hierarchical cluster analysis, participates of the new trend for WQI development, which uses rational, rather than subjective assessment. The GWQI is a successful index due to its ability to represent the groundwater quality of the state of Bahia, using a single mathematical formulation, the same five parameters, and unique weight for each parameter.

## Introduction

Many reviews about water quality indexing have been published by a variety of authors. For instance^1^, reviewed WQIs developed from 1960 to 2010;^2^ analyzed the performance of 30 existing WQIs;^3,4^ applied 7 different WQIs for, respectively, thirteen and sixteen months monitoring data in river waters; and^5^ reviewed 40 existing WQIs. The following statements were extracted from their conclusions: (i) although many WQIs are available, there is still a need of an overall WQI, able to incorporate the available data and describe the water quality for different uses; (ii) significant discrepancies were observed in classification from different methodologies; (iii) the most challenging aspect is that WQIs are developed for a specific region, being source-specific; (iv) no single WQI has been globally accepted; (v) there is no worldwide accepted method for implementing the steps used for developing a WQI; (vi) there is a continuing interest to develop accurate WQIs that suit a local or regional area; (vii) some future directions are still necessary due to the limitations of worldwide developed WQIs. The conclusions from these authors indicate the desire in finding a globally accepted water quality index, and, a method of development. However, so far, these objectives were not achieved.

The authors^6^ developed a critical review of the published literature on water quality indexing, up to 2020, working with 2049 articles from a variety of scientific journals. They used a three-stage sequential process of examination (bibliometric, scientometric, and qualitative valuation) identifying the most influential journals, researchers, articles, and countries dynamic in the research field of water quality indexing. Their scientometric analysis indicated that water quality indexing fills four classes: specific indices, human intervention, performance assessment, and emerging technologies. Moreover, the review of^7^ indicated that the WQIs are classified into four categories depending on the water end-use: (i) no specified end-use (WQI has a holistic view of the water); (ii) uses for drinking, irrigation, or industrial activities (WQI is highly target-specific); (iii) if the primary focus is management and planning of water resources (WQI has planning and management features); and, (iv) if statistical and mathematical models help to determine the overall water body health (WQI is a mathematical tool).

WQI, as a mathematical tool, has the goal to transform a variety of water quality parameters into a single value to describe the quality of a water body^2,8,9^. The development of the majority of the numerical WQIs involves the transformation of parameters with different units and dimensions, into dimensionless scale, defining subindices, and choosing different aggregation methods to generate the single value for the index^8^.

The first reported numerical index, target-specific, was proposed by^10^, to assess pollution reduction programs in rivers. Then, in 1970, emerged other important analytical index for surface water quality evaluation, the WQI-NSF from^11^, which is applied worldwide as originally proposed^12–15^, or modified, and renamed before application^16–22^, to cite a few. The WQI-NSF was proposed with the support of US National Sanitation Foundation, to express the surface water quality, using nine parameters associated to domestic wastewater pollution. The calculation involves transforming chemical concentrations values, in dimensionless quality grades, using normalized curves. The multiplicative formula to produce a single WQI-NSF value, operates the dimensionless subindex raised to a power, or the weight of importance of each variable. More recently, in 2001, emerged the WQI-CCME^23,24^, a statistical index to assess the quality of surface waters, very well-known and applied worldwide as it was proposed^3,4,13,14,25–35^; also applied after receiving some adaptation for local conditions^36,37^. The index^23^ was proposed with the support of the Canadian Council Minister of Environment, with the following characteristics: it is independent of dimensionless sub-indices; can incorporate from four to all measured parameters; all parameters had the same degree of importance, and had to be measured during four monitoring campaigns. Later^24^, changed one condition, which was: to incorporate, from a minimum of eight, to a maximum of twenty measured parameters.

The authors^38^ considered that, for the proper use of the WQI- CCME, it is necessary to define the time period for water quality evaluation, the choice of variables to use, and the objectives for the index calculation, as the factors (F1, F2) that compose the index, can vary when few variables are used or when the variables are closely related. The authors^3,4^, investigating seven frequently used indexes, found that the WQI-CCME was the most appropriate, for being conservative (indicated a stricter river water quality), and sensitive to changes in water quality. Moreover, the majority of authors that applied the WQI- CCME, favor it as the most flexible, because it can incorporate any parameter site specific; has an ease formulation; and can be easily adapted to legal requirements of different locations and different water uses.

In the meantime, since the 70’s, a variety of WQIs with planning and management characteristics, were developed in many countries, such as: for river pollution evaluation^39–42^; for public water supplier^43^; for river water quality status definition^44,45^ and others, such us, the Florida stream water quality index—SAFE^46^, the Lower Great Miami watershed enhancement program—WEP^47^, and the British Columbia (BC) water quality index^48^, to cite a few. All these indices have been evaluated in the literature, and, for all of them, are indicated some limitations in their ability to unequivocally represent the water quality. For instance^49^, investigated the WQI-(SAFE, WEP, and BC) considering the indices with too many variables, which, for most watersheds, are rarely found in a continuous manner. They found the WQI-BC with too many water use objectives: drinking, recreation, irrigation, livestock watering, wildlife, and aquatic life; each one, with different set of parameters and specific rankings. Also^49^, considered the WQIs’ formulas inefficient to evaluate the degree of pollution or the actual water quality in a stream. Then, they developed an analytical new index, using fewer variables and independent of standardized variables. However, they considered their new index with a limitation, as it could not be applied downstream of a wastewater treatment plant or in watercourses with large amounts of untreated human or animal waste. Finally, they concluded that, their new index gave results very similar to the WQI-NSF and WQI-WEP.

Recent development of WQIs for surface water, occupy the category of emerging-technologies, as they are based on mathematical approaches such as: multivariate statistics^50^; fuzzy inference system—FIS^51–53^; probabilistic neural network—PNN^53^; and artificial neural network—ANN^54^. About the development of the WQIs using emerging-technologies, the following conclusions were reached by these authors: (i) using statistical techniques reduce bias and it is more objective; (ii) multivariate statistics is more economic, as it identifies the significant parameters, reducing the time effort, and cost requirement, to monitor large number of variables; (iii) application of fuzzy techniques could interpret complex conditions in a river system, also, was appropriate to address uncertainty and subjectivity in environmental problems; (iv) fuzzy-logic-based methods may be useful to develop a water quality management strategy; (v) jointly application of fuzzy inference systems (FIS), Bayesian networks (BN), and probabilistic neural network (PNN) to the output of the WQI-NSF and WQI-CCME for river water, produced an accurate probabilistic water quality assessment; and, (vi) artificial neural network (ANN) using globally accepted parameters was successful in crating a WQI for surface water. Despite the fact the WQIs developed with emerging-technologies were all tailored to local or regional applications, the main feature was the absence of subjective assessments, as they derived from water quality datasets and specific mathematical correlation between variables. Thus, the emerging-technologies can provide methods with global application to develop WQIs for surface waters.

Regarding the studies on groundwater quality indexing, the author^6^ found that the WQIs are mainly in the class of water-use specific, as the primary regions of focus are those facing scarcity of surface water, thus, depending on the local aquifers to meet their water demands. These water-use specific WQIs are focused on assessing the hydrogeology of the study area, mainly for drinking and irrigation purposes^55–59^. However, the literature reports initiatives to communicate groundwater quality in the category human-intervention, performance-assessment. For instance^60^, developed the index SEQ-Eaux Souterraines with the support of the French Ministry of Waters, based on two notions: the ability of a water to satisfy a chosen use; and the alteration of water quality due to pressures from human activities. The SEQ-index uses a large number of parameters organized in seventeen groups of alteration, associated to the uses: drinking water, industrial, energy, irrigation and animal feed. It generates sub-indices for each group of alteration, and, the final value of the SEQ-index corresponds to the lowest value attributed to the set of sub-indices.

On the other hand, a variety of groundwater quality indices were derived to help policy makers and stakeholders, regarding the planning and management of groundwater resources. Many indices were derived from WQIs originally developed for surface waters. For instance, the WQI-CCME, due to its statistical formulation and flexibility of parameters selection, was adapted by many authors for groundwater quality evaluation^55,56,61–68^. Others, adapted the WQI-NSF, after identifying the most significant parameters for the groundwater quality evaluation and their degree of importance^68,69^. The work of^70,71^ used the mathematical formulation for the WQI-NSF to derive a groundwater quality index—NGWQI for the state of Bahia, Brazil. The NGWQI development involved the following steps: (i) subjective assessment for choosing the representative variable of the state of Bahia groundwater quality (hardness, chloride, fluoride, nitrate, total residue, and pH); (ii) subjective assessment to define the degree of importance of each chosen variable; (iii) development of normalized curves of concentration (c_i_) versus grades (q_i_), using the limits from Brazilian drinking water legislation to set the quality range from 0 (worst) to 100 (best); (iv) transformation of the physicochemical values in dimensionless subindices using the normalized curves; and, (v) calculation of the single value for the NGWQI based on the product of each grade (qi), raised to its weight (wi), or the degree of importance. The spatialization of the NGWQI in the four hydrogeological domains of the state of Bahia: sedimentary, crystalline, karstic and metasedimentary, was considered with good correlation with the groundwater quality, by hydrogeologists from CERB, the governmental drilling well company.

For groundwater applications, the trend of WQI development in the category of emerging-technologies, has also grown. In this category, can be cited: multivariate statistics and regression models^72^; multivariate statistics, probability curves and GIS^69^; regression models^73^; fuzzy methodology and GIS^74^; artificial neural network—ANN and multiple linear regression—MLR^75^; and entropy information theory^76–81^. The following conclusions were reached by these authors about the development of these WQIs: (i) jointly application of correlation analysis and multivariable linear regression helped to identify the sources and factors affecting the groundwater pollution of an urban aquifer. The regression model derived for the groundwater quality prediction was reliable and stable; (ii) probability curves defined the critical variables, and PCA determined the principal water quality parameters and their weights, to compose the WQI; (iii) regression models allowed predictions about past, present, or future groundwater quality events, in a less expensive manner, either in terms of time and/or money; (iv) hybrid WQI model, fuzzy-GIS-based, using seven critical parameters, was more reliable and pragmatic for groundwater-quality assessment and analysis at a larger scale; (v) jointly application of ANN and MLR models predicted precise values for a WQI, with sensitive performance for two seasons; (vi) information entropy methods avoid personal judgments about the weight of the parameters to participate in the WQI; (vii) entropy weighted water quality index (EWQI) has been recognized as the most unbiased model for assessing drinking water quality. Based on these comments, the WQIs developed for groundwater quality applications, were all tailored for local or regional situations. However, the most important feature of using the emerging-technologies is the absence of subjective assessments, as they derive from water quality datasets and specific mathematical correlation between variables. Similarly to surface waters, the emerging-technologies can provide methods with global application to develop WQIs for groundwater resources.

The present work develop a groundwater quality index—GWQI for the state of Bahia, Brazil, in the category of emerging-technologies, using the multivariable techniques: factorial analysis (FA), principal component analysis (PCA), and hierarchical cluster analysis (HCA), for parameter selection and determination of the degree of importance of each parameter. The method was totally rational, independent of subjective assessment, participating of the new trend for WQI development.

## Study area: state of Bahia, Brazil

### Geology and hydrogeology of the state of Bahia

**Figure 1:**
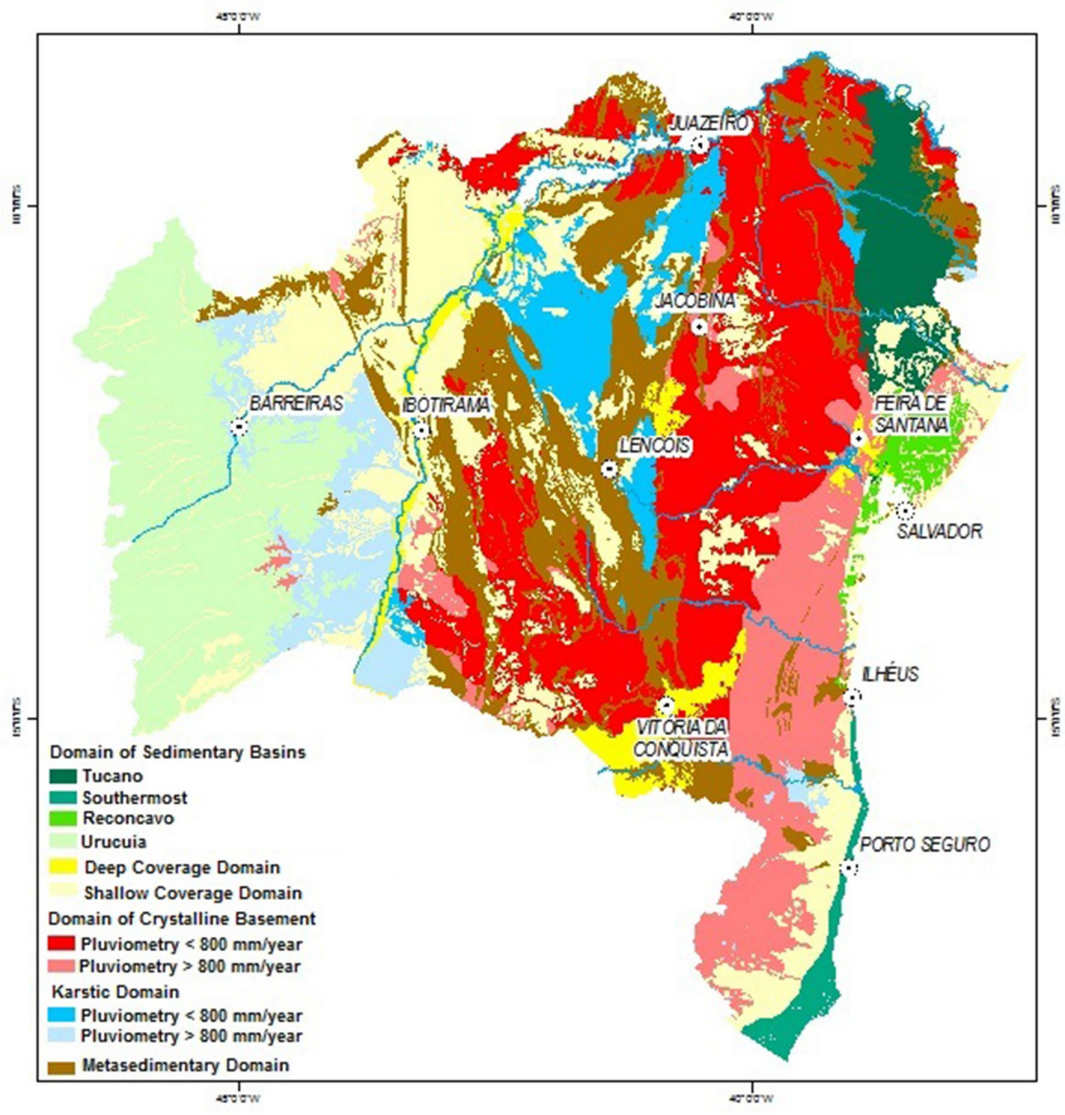
Geological and hydrogeological domains of the State of Bahia. Source: ^82^ modified from^83^, using ArcGIS version, 8.3^84^.

The study area is the whole state of Bahia, a federative unit in the Northeast Region of Brazil. The state of Bahia is approximately located between the coordinates 38ºE to 46ºW of longitude, and 9ºN to 17ºS of latitude, with an area of 567.295 km^2^, being the largest northeastern state in terms of land area, and fifth in the national ranking^82^. Figure 1 presents the map of the hydrogeologic domains of the State of Bahia^82^, modified from^83^, indicating the presence of eleven domains with the respective lithologies.

The Fig. 1 shows that the state of Bahia has great geological and hydrogeological diversity. In the coastal region, east of the state (18 to 65 km wide), occurs from north-to-south the sedimentary basins (Tucano, Reconcavo, and Southernmost). Next, emerges from north-to-south the crystalline domain (rainfall < 800 mm/year; and > 800 mm/year), plus detrital covers (shallow) at the south. In the central area occurs the karstic domain (rainfall < 800 mm/year), almost surrounded by the metasedimentary domain, plus detrital covers (deep) at the south. Next, towards the west, comes the sequence: detrital covers (shallow); karstic domain (rainfall > 800 mm/year), and the sedimentary basin (Urucuia). Predominantly, the domain of sedimentary rocks are composed by sandstones; the crystalline domain, by mafic and ultramafic iron producing rocks; the karstic domain, by carbonate rocks, limestones and pure quartzites; and the metasedimentary domain, also presents iron producing mafic and ultramafic rocks.

The state of Bahia hydrogeological characteristics are controlled by the factors: geology, climate, and precipitation. Consequently, the groundwater resources has a very heterogeneous geographical distribution. The authors^83^ delimited areas of similar hydrogeological behavior and groundwater production (Fig. 1). In the state of Bahia, the climate and precipitation have the following distribution: coastal region (humid; 1400–2600 mm/year); next stripe parallel to the coast, also at the western region (humid to sub-humid; 1000–1400 mm/year); following stripe parallel to the coast, also at the center, in addition to the karstic terrains in high topography (sub-humid to dry; 800–1200 mm/year); terrains of crystalline and karstic domains at the center/north (semiarid; ≤ 600 to ≤ 800 mm/year); far north (arid; 300–500 mm/year); far western region, in the stripe of 20- 80 km wide, (humid to sub-humid; 1300–1600 mm/year).

The Table 1 presents a description of the geological characteristics of the hydrogeological domains of the State of Bahia, and some aquifer characteristics: groundwater productivity and quality, from^85^ and^86^.

**Table 1:** Characteristics of the hydrogeological domains of the State of Bahia.

Domain	Description	State area %	Lithology and aquifer characteristic
Sedimentary basins	State eastern region: Reconcavo basin Rainfall rate: 1400 to 2600 mm/year	6.9	Predominance of sandstone. Sergi formation: small recharge area, variable flow rates and tendency of salinization in depth Marizal-São Sebastião formations: large recharge area, wells with large capacity: up to 450 m deep; flow rates up to 350 m^3^/h. Store water of good quality in the sandstones
	State eastern region: Tucano basin (South, Central, North) Rainfall rate: 1400 to 2600 mm/year		Predominance of sandstone but with lithological aspects (layers of shale and carbonates) with variable groundwater favorability. Marizal, Sergi, Aliança formations: store water of good quality. However, the Marizal presents salinization in depth. Group Ilhas presents less groundwater favorability
	State eastern region: Southernmost basin. Rainfall rate: 1000–1400 mm/year		Quaternary coastal deposits and Tertiary-Quaternary Barreiras Formation. Averaged flow rate of 27 m^3^/h. Store water of good quality in the sandstones
	State western region: Urucuia basin Rainfall rate: 1300–1600 mm/year	16.3	Predominance of Sandstone. State largest groundwater reserve; high potentiality; excellent groundwater quality
Sedimentary coverage	Shallow coverage (dune and alluvial sands); Deep coverage (Barreiras Formation) Rainfall rate: 800–1200 mm/year	15	Dune and alluvial sands and clayey sand sediments. The shallow cover has reduced depth and store pluvial waters. Aquifer of high vulnerability. The deep covers has wells with 150 m deep, flow rates greater than 50 m^3^/h. Both sediments stores water with good quality
Crystalline Domain	State central-eastern portion from north to south Rainfall rate: > 800 mm/year	34.3	Granulites; basalt and gabbro; plutonic bodies Shallow, fissural or fractured free aquifers, low storage capacity, low permeability, heterogeneous and anisotropic. For rainfall > 800 mm/year, average flow rates is 9.9 m^3^/h, and the groundwater has from regular to inferior quality
	State north central part Rainfall rate < 800 mm/year		Granodioritic and granitic rocks, mafic–ultramafic and calcium-silicate rocks For rainfall ≤ 800 mm/year, average flow rates is 9.1 m^3^/h, and the groundwater presents generally inferior quality
Karstic Domain	State western and coastal southern region Rainfall rate: > 800 mm/year	13.2	Carbonate rocks, and glaciogenic sediments. Dolomitic marbles limestones, and pure quartzites. Free fissural aquifers of high heterogeneity and anisotropy. Due to a better rainfall rate, the averaged flow rate is 4.0 m^3^/h, and the groundwater has from regular to inferior quality
	State central and north-northeast region Rainfall rate: < 800 mm/year		Glacial diamictites, and carbonate from shallow marine and tidal plain Limestones and dolomites. Free aquifers of high heterogeneity and anisotropy, with low storage capacity. Due to the low rainfall rate, the averaged flow rate is 3.4 m^3^/h, and the groundwater has generally inferior quality
Meta sedimentary Domain	Mountain range from Jacobina and Chapada Diamantina Rainfall rate: 800–1200 mm/year	14.3	Quartzites, metarenites, sandstone, claystone; carbonous phyllites, mafic–ultramafic rocks in the north–south direction; and Greenstone Belts Free aquifers of fissural and fractured nature, of high permeability, high recharge rate. Averaged flow rate of 6.9 m^3^/h, and the groundwater has from regular to inferior quality

### State of Bahia Grouwndwater Quality

This section presents the groundwater quality of the state of Bahia, per hydrogeological domain, summarized in Table 1. Also discuss the results published by^85,86,88^, to address the importance of the parameters (hardness, total residue, sulphate, and iron), selected to be part of the GWQI. The work of^85^ presents the average values for (chloride, total hardness, total residue, and nitrate) in the groundwater of the state of Bahia, whose limits for drinking water^87^, are, respectively, (250 mg/L; 500 mg/L; 1000 mg/L; and 10 mg/L). The work of^86^ studied the parameter iron in the groundwater of the state of Bahia, based on 5583 wells drilled in the period (2003–2013). He found 978 wells (17.5%), with high iron content (> 0.3 mg/L), the limit for drinking water^87^. The work of^88^ studied the parameter sulphate in the groundwater of the state of Bahia using the same data base from^86^. She found from 2792 wells, 289 (10.4%) with high sulfate concentration (> 250 mg/L), the limit for drinking water^87^. The predominant species with high sulfate concentration were (CaSO_4_ and MgSO_4_), smaller quantities for (NaSO_4_) and very low for (KSO_4_). She found that aquifer geology, and not rainfall, was the most influential on sulfate concentration and species.

From^85^, the deep sedimentary coverage presents average values for (chloride, total hardness, total residue, and nitrate) below the limits (groundwater of good quality); nevertheless, due to its shallowness or not so deep layers, it presents high vulnerability to contaminants.

In general, the sedimentary basin has predominance of sandstone with water of good quality, however, areas with small recharge and variable flow rates has tendency of salinization in depth. The Tucano sedimentary basin presents some lithological aspects (layers of shale and carbonates) that favor the occurrence of groundwater with variable quality. From^85^, the sedimentary basins present, in general, average values for (chloride, total hardness, total residue, and nitrate) below the limits (groundwater of good quality), only the (Sergi/Aliança formations) presents chloride slightly above the limit.

The fractured crystalline aquifer presents unfavorable water circulation, thus has generally water of inferior quality. From^85^, the crystalline domain presents average values above the limits, for three parameters: chloride, total hardness, and total residue (groundwater with quality regular or poor); while for nitrate, the average values are below the limit, indicating not significant human impact. From^86^, the mixed sedimentary/crystalline aquifer had (46.9%) of wells with high iron content, due to the presence of iron producing rocks in the crystalline portion, and larger water circulation in the sedimentary portion.

The karstic domain, due to the presence of carbonates, only presents water of better quality in places were the rainfall rates are favorable. From^85^, the karstic domain (> 800 mm/year) presents the average values for (chloride, total hardness, total residue, and nitrate) below the limits (groundwater of good quality), clearly related with the larger rainfall; while the karstic domain (< 800 mm/year) presents average values above the limits (groundwater with quality regular or poor). From^86^, the karstic domain had the smallest percentage of wells with high iron content (9.88%).

The metasedimentary domain with free aquifers of fissural and fractured nature, associated with a variety of lithological and geological characteristics, presents groundwater from regular to inferior quality. From^85^, for the metasedimentary domain, only the parameters, total hardness and nitrate, present average values below the limits, indicating varying groundwater quality.

For nitrate^85^, found, only in the karstic domain (< 800 mm/year) an average value (10.7 mg/L) slightly above the limit established for drinking water (10 mg/L). Nitrate is an anthropic groundwater chemical parameter derived from fertilizers (the karstic domain has extensive agricultural activities), and from domestic wastewater (the urban area uses septic tanks and has inadequate sewer system). The presence of nitrate in the aquifer of karstic terrains is also favored by the presence of caves and dolines.

### Geostatistic applied to parameters of groundwater quality of the state of Bahia

For the parameters, chloride, total hardness, total residue, and nitrate in the hydrogeological domains of the state of Bahia, the work of^85^ developed semivariograms, a geostatistical tool to investigate how much the variable is regionalized, which characterize a natural phenomenon^89,90^. In the semivariogram function, the parameter (a) represents the maximum distance at which the variables correlate with themselves.

A regionalized variable is indicated by a spatial correlation structure, or, a function [Z(x)] for each point (x) in the space n dimensional (R_n_), presenting two characteristics: randomness, or erratic variations; and structure, or the global aspect of the regionalized phenomenon. To study spatial and temporal variability of a given property, the geostatistic may assist in identifying the most probable spatial patterns of a parameter distribution^91,92^. The literature presents a variety of geostatistical tools that allow estimating the probability of occurrence of a given event, in places not investigated, from information obtained elsewhere^93,94^. When samples are collected in the field, it is necessary, before to proceed an interpolation between two measured locations, to build up isoline maps with the appropriate tool to establish the spatial dependence. The semivariogram indicates the most appropriate spatial dependency function of the variable under study^89^. Once the semivariogram is known and the spatial dependence is confirmed, values can be interpolated at any position in the field of study, and the interpolation method is called Kriging^93,94^.

From the work of^85^, the variables (chloride, total hardness, and total residue) present the parameter (a) with values (204.3; 236.9; and 170.7 km), respectively, indicating that these are regionalized variable. For nitrate, the parameter (a = 4.95 km), a relatively small distance, after which the nitrate values no longer correlate, and this is not a regionalized variable. The spatialization of nitrate values in the groundwater of the state of Bahia by^85^, indicated high nitrate concentrations in the most vulnerable areas of the karstic and crystalline aquifers, due to three main factors: shallow aquifers; karstic and fractured structures; and vectors of pollution (irrigated agriculture and domestic wastewater effluents).

## Material and methods

### Selection of wells and grouwndwater samples for statistical analysis

The database from the state of Bahia well drilling company, CERB –Water Resources and Environmental Engineering Company^95^, provided a comprehensive amount of data for the hydrogeological domains of the State of Bahia. The physicochemical analysis were developed at LABDEA, the laboratory of the Environment Engineering Department, Polytechnic School of the Federal University of Bahia (UFBA).

A total of 600 from 1969 wells, were used to apply the statistical analysis and develop the groundwater quality index—GWQI. The remaining 1369 wells were used to apply the GWQI and to test the model adequacy to describe the state of Bahia groundwater quality. The Table 2 presents for both sets (600 and 1369 wells), the statistics for the number of wells and municipalities envolved, per hydrogeological domain, considered here, as criteria to guarantee the sample randomness.

**Table 2:** Number of wells and municipalities per hydrogeological domain, and related percentages, for the set of 600 and 1369 wells.

Hydrogeological domain	For the set of 600 wells			For the set of 1369 wells			Ratio of municipalities N_600_/N_1369_
	Number of wells	Percentages of wells %	Number of municipalities	Number of wells	Percentages of wells %	Number of municipalities	
Sedimentary	113	18.8	48	327	23.9	70	0.686
Crystalline	261	43.5	128	614	44.8	147	0.871
Karstic	135	22.5	46	212	15.4	58	0.793
Metasedimentary	91	15.2	40	217	15.9	60	0.667
Totals	600	100.0	262	1369	100.0	335	0.782

For the total of 1969 wells, the number of wells per hydrogeological domain is: Sedimentary (440 or 22.4%), Crystalline (875 or 44.4%), Karstic (346 or 17.6%), and Metasedimentary (308 or 15.6%).

From Table 2, the percentages verified in the set (600 wells) are not exactly the same, as those in the set (1369 wells), however, they are close enough to guarantee the similarity. For instance, 78.2% of the total number of municipalities in the set with 1369 wells (335), it is present in the set of 600 wells (262), indicating good areal distribution of the wells. Classifying per hydrogeological domain, it is verified that, from 66.7 to 87.1% of the number of municipalities in the set (1369 wells), it is present in the set of (600 wells), indicating good hydrogeological representativeness. Thus, the sample of 600 wells can adequately represent the total.

The data bank with the 600 wells was submitted as Supplementary Material. Also, was submitted the data bank with 1369 wells, including the necessary information to calculate the GWQI and the previous index NGWQI, for comparison. These two spreadsheets present summary tables and statistical results discussed in the paper.

### Multivariable statistical methods

Multivariable analysis are largely applied to environmental data, seeking to identify the significant parameters from a large data set of multiple variables^22,96–102^. To identify the factors responsible for the groundwater pollution in a shallow urban aquifer of Yan’an City, in China^72^, used the methods of principal component analysis (PCA), hierarchical cluster analysis (HCA), and multivariable linear regressions (MLR) to search the relationships between the groundwater quality parameters and to generate a regression model. Also, in China^103^, used multivariate analysis to understand the hydrogeochemical processes occurring in the water of the Guohua phosphorite mine. In India^104^, used these techniques to elucidate aspects of the groundwater geochemistry and drinking water suitability in the Kudal region. In Brazil, state of Bahia^105^, applied multivariable analysis for groundwater quality evaluation in the central-southern portion of the state, while^106^, used to classify the groundwater quality in the Salitre river watershed. And, in the state of Ceará^107^, used to explain the processes responsible for the groundwater quality in the city of Fortaleza; while^108^, were searching the similarity of hydrochemical variables in the Salgado river watershed.

The multivariable methods applied in this work were factorial analysis (FA); the principal component analysis (PCA) and the hierarchical clustering analysis (HCA). The factorial analysis was used to define the structure of the variables correlations^109^. The (FA) calculates the correlation matrix between variables, it does the extraction of initial factors and does the rotation of the matrix^109^. The correlation matrix allows to indicate the similarities and differences in the cluster analysis^110^.

The method of principal component analysis (PCA) helps to extract the factors from the correlation matrix, necessary to explain the covariance structure through linear combinations of the original variables^111,112^. The (PCA) reduces the total number of variables to a smaller data set of statistical variables, while preserving the variability with a minimal loss of information. Each factorial load represents the degree of contribution of the variable to the formation of the factor. The variables with the highest factorial load are considered of greater importance and should influence more on the factor label^109^. The PCA also helps to detect through communalities, how much each parameter explains each factor^113^. The normalized Varimax rotation, an orthogonal rotation of the factors, helps to minimize the number of variables with high loads in different factors.

The hierarchical cluster analysis (HCA) has the goal to produce the variables hierarchical classification, necessary to detect the most pertinent properties to be included in the index. The (HCA) build the tree diagram where the most similar properties in the study are placed on branches that are close together^109^. The clustering was performed using the method of^114^, which creates a small number of clusters with relatively more properties. The cluster analysis define the similarities and dissimilarities between variables through a dendrogram. The key to interpreting a dendrogram is to look at the point at which any given pair of properties join together in the tree diagram. The pair that join together sooner are more similar to each other than those that join together later. In the present work, the (HCA) helped to detect the most pertinent properties to be included in the groundwater quality index—GWQI.

## Results and discussion

### Application of the multivariable analysis to develop the Gwqi

The sample with 600 wells was a satisfactory number to apply the multivariable analysis, according to the simplified approach from^109^. For these authors, the number of cases for the factorial analysis must be at least 5 times the number of measured variables. The number of measured variables indicated in the CERB database was (26), then, (5 × 26 variables = 130). Thus, the sample of 600 wells was representative of the 1369 wells used to test the index, besides it was a random sample, as demonstrated in the topic 3.1.

The results from the exploratory analysis are on Table 3. It involves the descriptive statistics (minimum, maximum, and average; the quartiles, lower, upper and median; standard deviation, standard error and confidence interval), calculated with the software Statistica, version 7.0^115^. From the 26 variables from CERB data bank, were excluded the variables considered not representative or presenting nonconformities: sodium (9 valid samples), potassium (7 valid samples), ammoniacal nitrogen (4 valid samples), and acidity (not representative). The Table 3 presents only the 22 variables that will be the input for the multivariable analysis.

**Table 3:** Descriptive statistics including the coefficient of variation (CV), with 22 representative variables.

n = 22	Valid number	Mean	Confidence interval − 95%	Confidence interval + 95%	Median	Minimum	Maximum	Lower quartile	Upper quartile	Standard deviation	Standard error	CV (%)
Depth	599	103.3	99.37	107.2	90.0	10.0	339.2	72.0	130.0	48.5	2.0	47
Static level	595	19.4	17.0	21.7	7.3	− 0.5	209.3	3.0	21.2	29.6	1.2	153
Dynamic level	595	53.4	50.87	55.82	52.24	1.63	220.46	35.24	62.75	30.75	1.26	58
Flow rate	595	9.2	8.3	10.1	5.1	0.1	88.0	1.9	12.0	10.9	0.5	118
pH	594	8.0	7.91	8.02	8.00	3.62	10.52	7.64	8.37	0.69	0.03	9
Turbidity	595	10.8	7.04	14.63	3.00	0.08	907.00	1.70	6.63	47.12	1.93	435
Conductivity	571	2804	2459	3148	1069	21	20,000	450	2880	4192	175	149
Color	581	8.0	6.7	9.3	5.0	0.0	160.0	5.0	5.0	15.9	0.7	199
Alkali-HCO	595	184.7	174.7	194.8	179.9	0.0	710.8	78.8	257.2	125.0	5.1	68
Alkali-CO	595	13.3	11.5	15.2	0.0	0.0	176.0	0.0	21.1	22.9	0.9	172
Alkali-OH	595	0.2	0.0	0.3	0.0	0.0	28.9	0.0	0.0	1.9	0.1	1233
Sulphate	573	108.5	91.7	125.3	27.5	0.0	2180.7	5.8	120.0	204.6	8.6	189
Chloride	599	855.8	708.1	1003.6	145.0	1.5	18,407.5	36.7	654.0	1841.3	75.2	215
Calcium	525	396.0	344.4	447.7	195.3	0.0	6006.0	54.4	454.9	602.4	26.2	152
Magnesium	525	134.4	110.0	159.0	31.7	0.0	2551.0	10.8	106.0	285.5	12.5	212
Nitrite	598	0.04	0.01	0.06	0.00	0.00	8.00	0.00	0.01	0.34	0.01	848
Nitrate	597	4.9	3.9	5.8	0.6	0.0	135.0	0.01	4.2	11.5	0.5	238
Iron	599	1.1	0.7	1.4	0.2	0.0	97.4	0.1	0.6	4.7	0.2	442
Silica	566	22.8	21.6	24.0	20.6	2.1	101.0	12.5	30.5	14.1	0.6	62
Fluoride	589	0.8	0.7	0.9	0.4	0.0	30.0	0.2	0.8	1.7	0.1	211
Hardness	551	958.5	813.9	1103.2	331.8	3.0	13,773.0	112.0	917.0	1728.8	73.7	180
Total Residue	600	2356	2010	2702	764	18	47,098	328	2162	4318	176	183

In Table 3, eleven variables present values equal to zero (0.0) as their minimum. These values resulted from the substitution of the laboratorial expression (below detection limit) by (zeros). These “not measured data” receive, in the literature, the designation of “censored data”. The authors^116^ discuss four different procedures for solving the censured data: substitution, parametric methods, robust methods and non-parametric methods, all of them, presenting advantages and limitations. They say that, the simplest method to replace the undetected values is using a constant value below the detection limit. However, any value between zero and the detection limit can lead to deviations in the descriptive statistics: zeros, tend to produce underestimated averages, and the detection limit, tends to produce overestimated averages. In Table 3, zeros replaced, systematically, the censored data. The impact of this choice was evaluated, calculating the averages for both extremes (zero and detection limit). The spreadsheet for 600 wells (Supplementary Material) presents the averages and standard deviations for the parameters iron, fluoride and sulphate (ones with the largest amount of zeros), showing small impact. Consequently, in this work, the substitution by zeros has no negative consequences.

**Figure 2:**
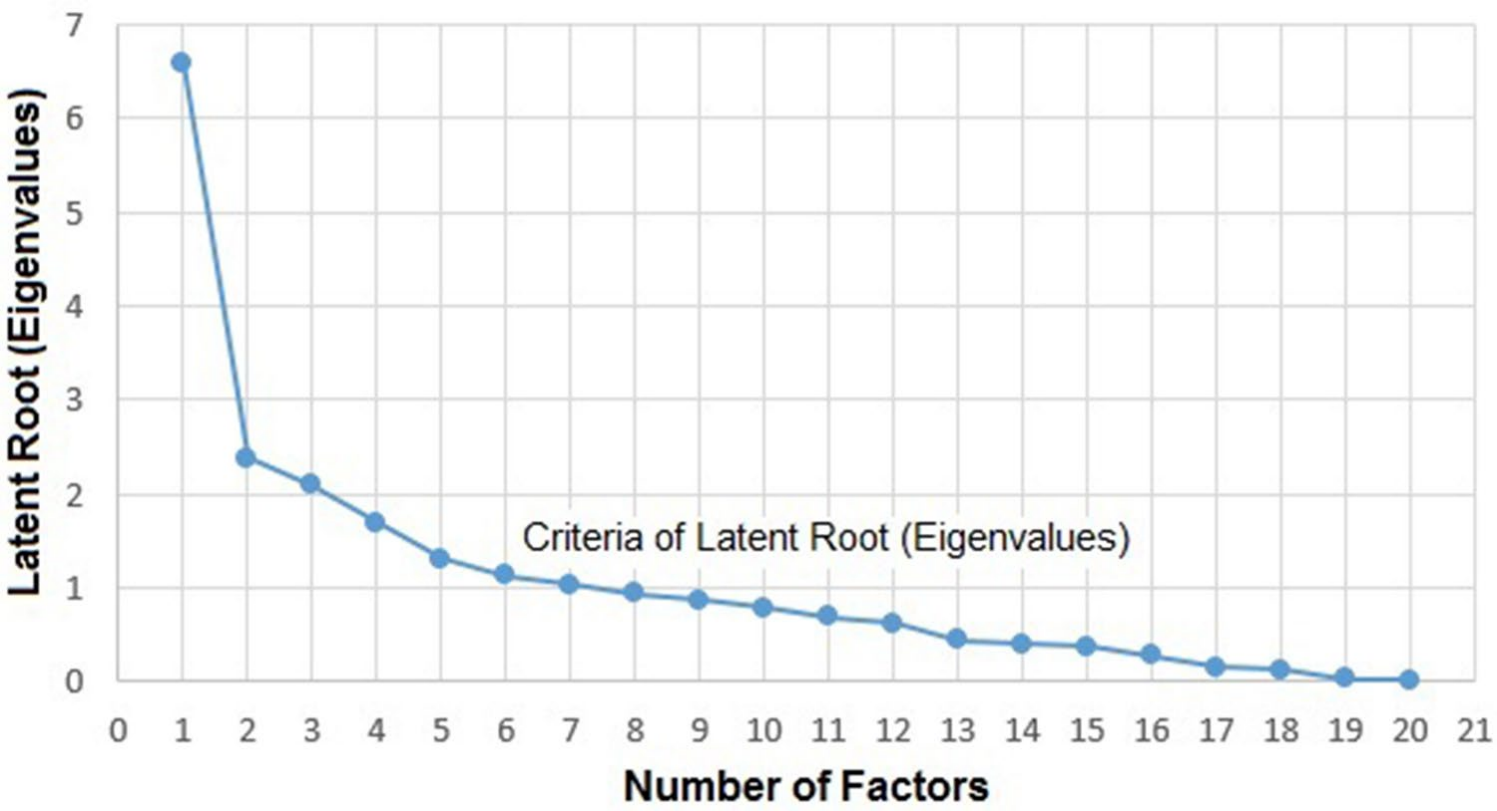
Screen test indicating the number of factors to be extract, using Excel, version 15.0^117^.

The multivariable analysis developed in this work, applied the methods of factorial analysis (FA), the principal component analysis (PCA), and the hierarchical cluster analysis (HCA), using the Statistica, version 7.0^115^. To identify the optimal number of factors to participate in the GWQI, Fig. 2 shows the criterion of the latent root. As recommended by^109^, only factors with latent roots or eigenvalues greater than one are considered significant. Figure 2 shows that the limiting value is 7 factors.

Another procedure to decide how many factors to participate of the GWQI, is the criterion adopted by^118^, which is, maintaining a minimum explanation of 60% of the cumulative variance. Table 4 presents the eigenvalues from the principal component analysis (PCA), the percentage of variance explained by each component; and the cumulative variance. The cumulative variance for five (5) factors, which is equal to 63.91%, satisfies the recommendation, and was adopted in the present work.

**Table 4:** Eigenvalues (Data Bank NUPEA 600 wells).

Value	Eigenvalue	% Total Variance	Cumulative eigenvalue	Cumulative %
1	6.581434	29.92	6.58	29.92
2	2.367200	10.76	8.95	40.68
3	2.103711	9.56	11.05	50.24
4	1.686792	7.67	12.74	57.91
5	1.322018	6.01	14.06	63.91
6	1.116483	5.07	15.18	68.99
7	1.018798	4.63	16.20	73.62

Extraction: Principal Components.

The Table 5 shows the matrix of factorial loads, after the Normalized Varimax rotation performed on the factorial axes. The factorial load is the correlation of the variable with the respective factor. If that load assumes a positive value, means that the variable has a positive correlation with the factor, and if it is negative, this correlation is negative, or, the variable has a direction of variation opposite to that of the construct. The Table 5 shows both results, positive and negative.

**Table 5:** Factorial Loads after Normalized Varimax rotation.

n = 22	Factor 1	Factor 2	Factor 3	Factor 4	Factor 5	Factor 6
Depth	− 0.081	− 0.810	0.039	− 0.106	− 0.372	− 0.010
Static level	− 0.121	− 0.862	− 0.131	− 0.014	− 0.076	0.029
Dynamic level	0.016	− 0.922	0.085	0.001	0.221	0.029
Flow rate	− 0.093	0.125	− 0.176	− 0.085	− 0.792	0.700
pH	0.139	− 0.017	0.110	− 0.132	0.074	− 0.500
Turbidity	0.150	− 0.016	− 0.055	0.814	0.034	0.002
Conductivity	0.944	0.051	0.078	0.037	0.132	0.022
Color	− 0.070	0.044	− 0.164	0.579	0.156	− 0.090
Alkali-HCO	0.286	0.081	0.597	− 0.112	0.169	0.314
Alkali-CO	− 0.142	− 0.040	0.698	0.009	0.153	− 0.236
Alkali-OH	− 0.019	0.052	− 0.118	− 0.079	0.044	− 0.561
Sulphate	0.741	− 0.020	0.134	− 0.107	− 0.018	0.086
Chloride	0.969	0.030	0.017	0.056	0.058	− 0.019
Calcium	0.940	0.049	0.011	0.051	0.074	0.058
Magnesium	0.959	0.041	0.019	0.028	0.057	− 0.018
Nitrite	0.052	− 0.153	0.008	− 0.035	0.057	0.222
Nitrate	0.133	0.155	− 0.093	− 0.253	0.119	0.572
Iron	0.036	0.081	0.198	0.613	− 0.146	0.134
Silica	0.129	0.189	− 0.001	− 0.032	0.694	− 0.062
Fluoride	0.128	0.006	0.702	0.009	− 0.048	0.014
Hardness	0.982	0.044	0.013	0.036	0.064	0.010
Total Residue	0.977	0.033	0.034	0.046	0.062	− 0.023

The recommendation from^109^ is that, factor loads with values above ± 0.50 are of practical significance, however, this work adopted a factor load higher than the minimum recommended value. For instance, from (Factor 4), the parameter iron, with factor load (0.613), was the minimum value considered significant in this work.

**Figure 3:**
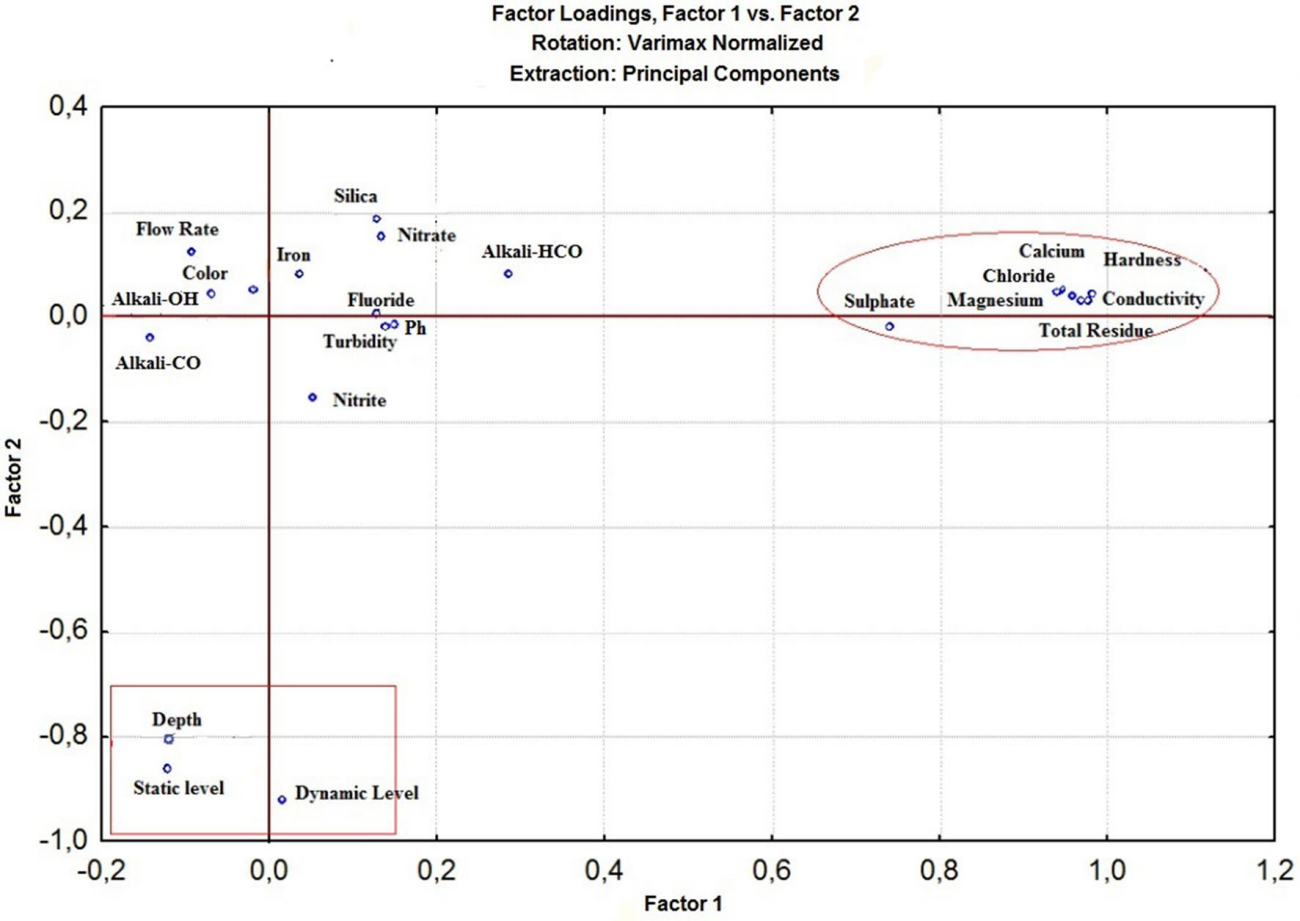
Graphical representation of factors F1 x F2 (Normalized Varimax Rotation), using Statistica, version 7.0^115^.

**Figure 4:**
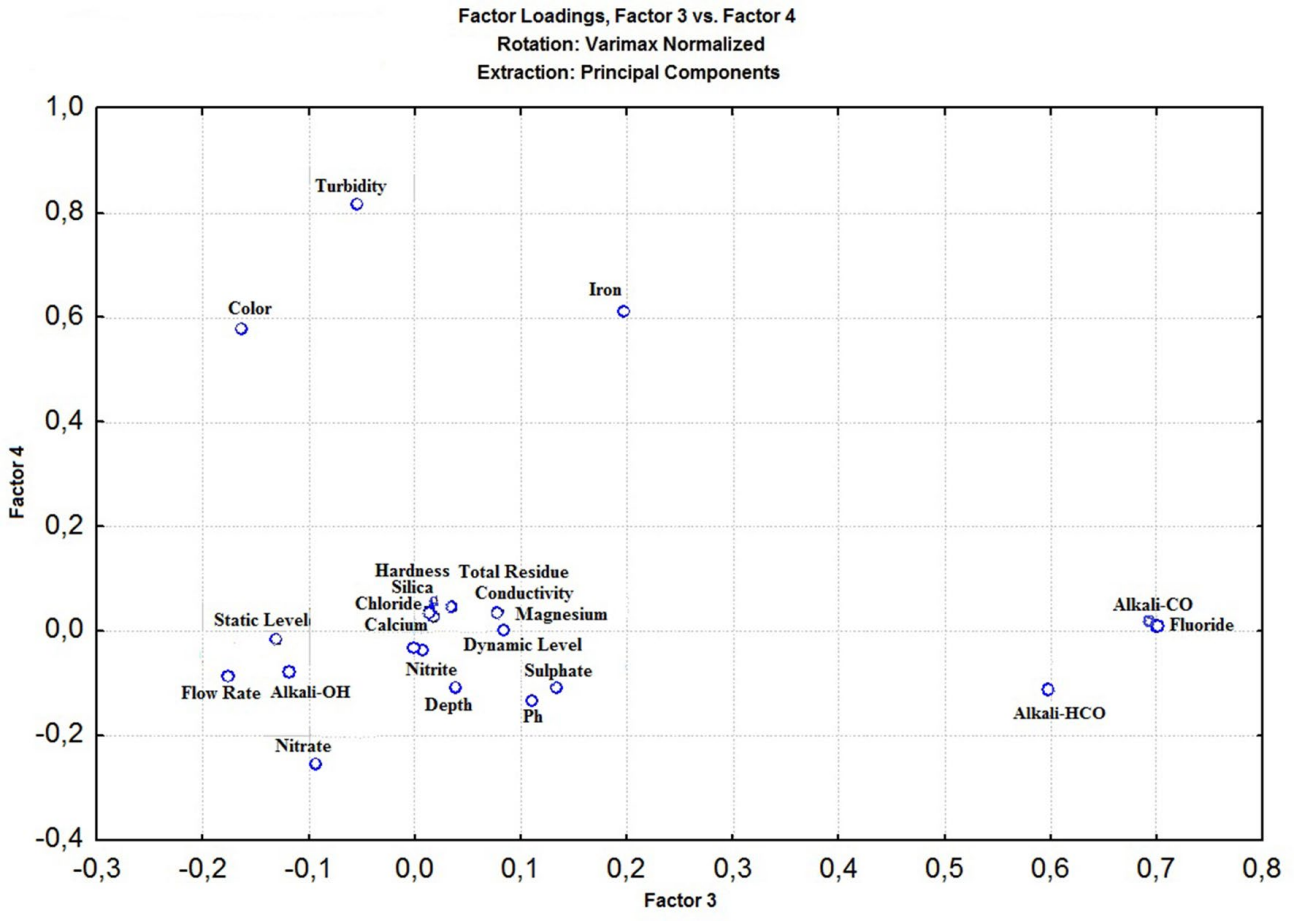
Graphical representation of factors F3 x F4 (Normalized Varimax Rotation), using Statistica, version 7.0^115^.

**Figure 5:**
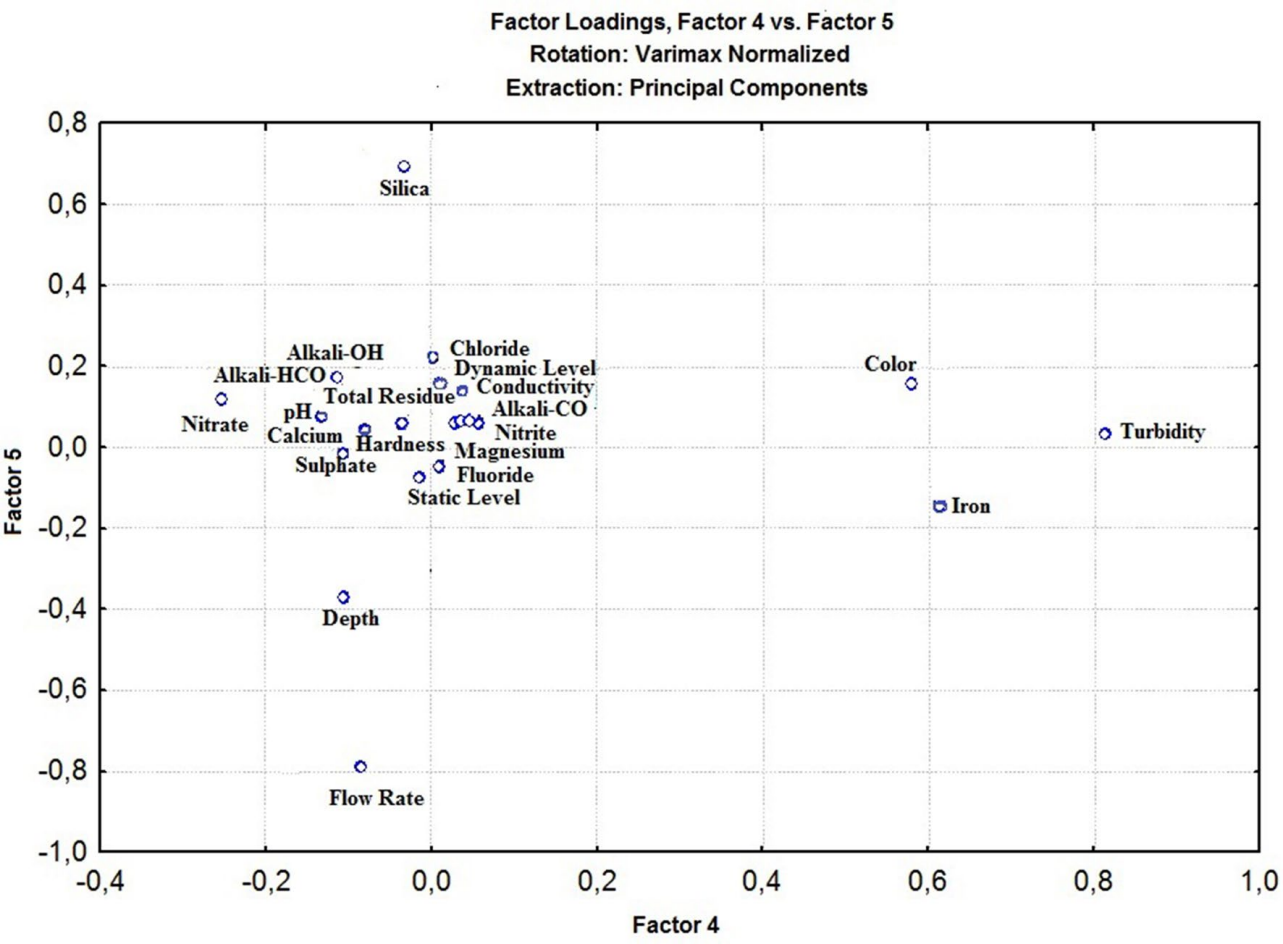
Graphical representation of factors F4 x F5 (Normalized Varimax Rotation), using Statistica, version 7.0^115^.

The application of the principal component analysis (PCA) helped to evaluate the variable level of explanation relevant to the analysis. Figures 3, 4, 5 show the graphical representations of the factorial plans: Fig. 3 (Factor 1 × Factor 2), Fig. 4 (Factor 3 × Factor 4), and Fig. 5 (Factor 4 × Factor 5).

In Fig. 3 (Factor 1 × Factor 2), the (Factor 1) explains 29.92% of the total variability of the data, and is the most important in the analysis, and (Factor 2), explains 10.76%, as shown on Table 7. From (Factor 1), seven (7) relevant variables related to water quality emerged with the greatest factorial load: calcium (0.940), chloride (0.969), conductivity (0.944), hardness (0.982), magnesium (0.959), sulfate (0.741), and total residues (0.977). From (Factor 2) emerged three (3) hydraulic variables with the greatest factorial loads: the dynamic level (− 0.922), static level (− 0.862), and depth (− 0.810). The total of significant parameters, so far, is ten (10).

In Fig. 4 (Factor 3 × Factor 4), the (Factor 3) explains 9.56% of the total variability of the data, and (Factor 4) explains 7.67%, shown on Table 7. From (Factor 3), the parameter fluoride has great significance (0.702), and from (Factor 4), turbidity has factor load (0.814), and iron (0.613). Turbidity was discharged because during a normal regime of groundwater exploitation this parameter is no longer significant in the water well. Thus, two (2) significant water quality parameters were identified, totaling twelve (12) significant parameters.

**Figure 6:**
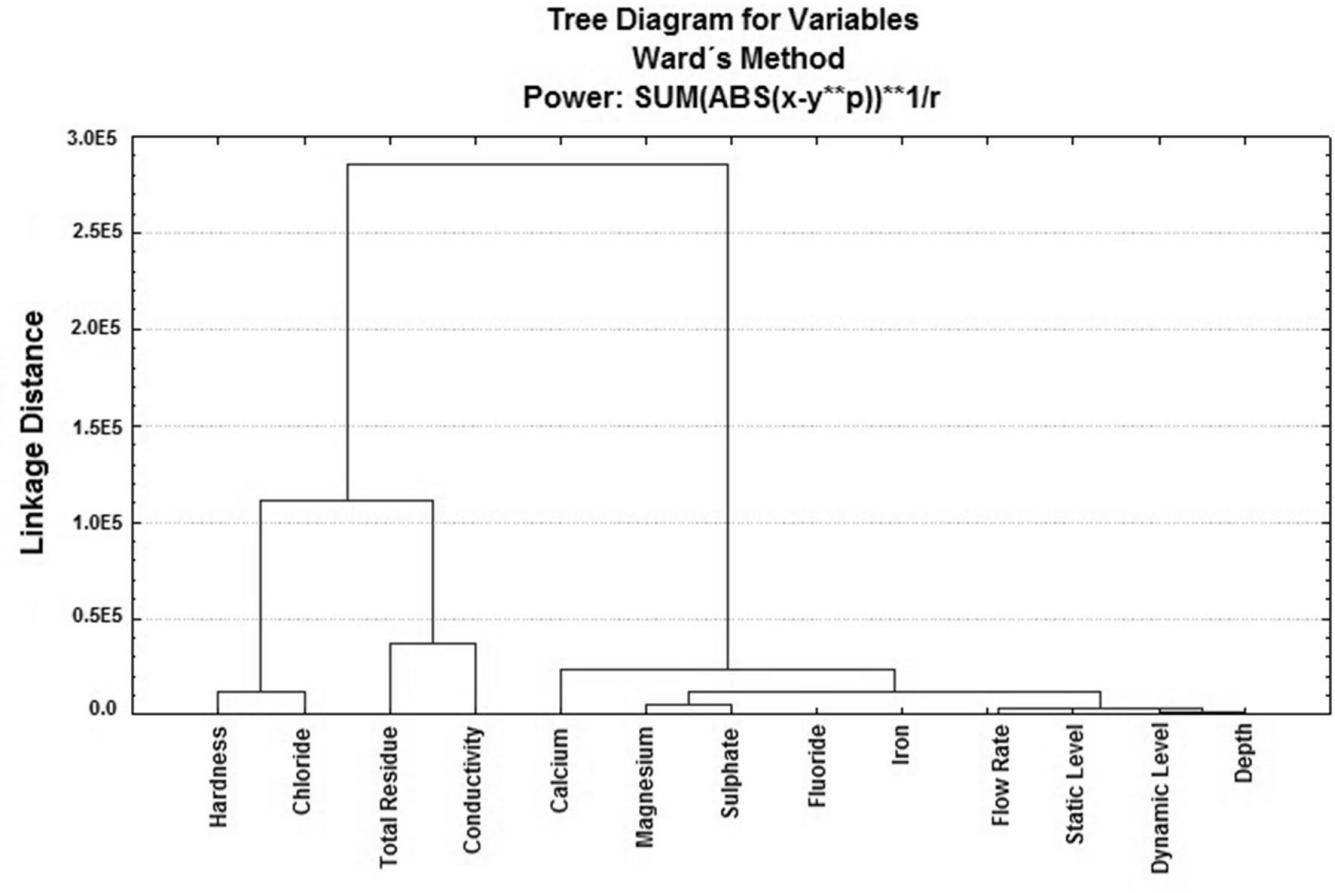
“R” mode dendrogram from the hierarchical cluster analysis, using Statistica, version 7.0^115^.

In Fig. 5 (Factor 4 × Factor 5), the (Factor 4) explains 7.67% of the total variability of the data, and (Factor 5) explains 6.01%, as shown on Table 7. From (Factor 5), the parameter flow rate with factor load (− 0.792) is the most significant. Consequently, the number of factors to be involved in the hierarchical cluster analysis is thirteen (13), with nine (9) related to water quality, and four (4) are hydraulic parameters. However, the hydraulic parameters, not related to water quality, will not be considered to compose the GWQI. Figure 6 presents the dendrogram from the hierarchical cluster analysis (HCA).

The dendrogram shows the formation of 3 groups of parameters with high internal similarity: “hardness x chloride”, “total residue x conductivity”, and “calcium x magnesium and sulphate”. This work choose only five (5) relevant variables, a total that responds for 63.91% of the total variance, satisfying the recommendation from^118^. The choices were: hardness (instead chloride, as they belongs to the same group); total residue (instead conductivity, as total residue is a chemical parameter); sulphate (instead calcium or magnesium, as both variables are present in hardness). In addition, were considered fluoride and iron, which are independent from each other. Thus, the variables to include in the GWQI to express the state of Bahia groundwater quality are: hardness, total residue, sulphate, fluoride and iron.

The next step for the GWQI formulation is, to define the degree of relevance of each parameter, in order to establish the relative weight (w_i_), necessary to the GWQI model. The starting point was to examine the communality values calculated after the normalized Varimax rotation, which represent the amount of variance explained by each variable in the factorial solution. The Table 6 presents the communality values (from 1 to 6 factors).

**Table 6:** Communalities (Data Bank with 600 wells).

n = 22	From1 factor	From 2 factors	From 3 factors	From 4 factors	From 5 factors	From 6 factors	Multiple R-square
Depth	0.007	0.662	0.664	0.675	0.813	0.813	0.661
Static level	0.015	0.758	0.775	0.775	0.781	0.782	0.634
Dynamic level	0.000	0.850	0.857	0.857	0.906	0.907	0.765
Flow rate	0.009	0.024	0.055	0.063	0.690	0.700	0.482
pH	0.019	0.020	0.032	0.049	0.055	0.305	0.041
Turbidity	0.022	0.024	0.026	0.689	0.690	0.690	0.286
Conductivity	0.892	0.895	0.901	0.902	0.919	0.920	0.904
Color	0.005	0.007	0.034	0.369	0.394	0.402	0.135
Alkali-HCO	0.082	0.088	0.445	0.457	0.486	0.585	0.362
Alkali-CO	0.020	0.022	0.509	0.509	0.533	0.589	0.166
Alkali-OH	0.000	0.003	0.017	0.023	0.025	0.340	0.027
Sulphate	0.549	0.549	0.567	0.579	0.579	0.587	0.529
Chloride	0.938	0.939	0.940	0.943	0.946	0.946	0.970
Calcium	0.883	0.885	0.885	0.888	0.893	0.897	0.963
Magnesium	0.919	0.921	0.922	0.922	0.925	0.926	0.985
Nitrite	0.003	0.026	0.026	0.027	0.031	0.080	0.033
Nitrate	0.018	0.042	0.050	0.114	0.129	0.456	0.123
Iron	0.001	0.008	0.047	0.423	0.444	0.462	0.136
Silica	0.017	0.052	0.052	0.053	0.536	0.539	0.213
Fluoride	0.016	0.016	0.509	0.509	0.511	0.511	0.164
Hardness	0.964	0.966	0.966	0.968	0.972	0.972	0.993
Total Residue	0.953	0.955	0.956	0.958	0.962	0.962	0.977

Extraction: Principal Component Analysis. Rotation: Varimax Normalized.

The largest communality value in the column (5 factors), is hardness (0.972), providing the greatest relative weight (w_i_). The others are: total residue (0.962), sulphate (0.579), fluoride (0.511), and iron (0.444). Then, on Table 7 it is demonstrated the procedure to obtain the weights (w_i_), based on the communality values for the five parameters (hardness, total residue, sulphate, fluoride and iron).

**Table 7:** Description how to obtain the relative weight (w_i_) of each parameter.

Parameters	Commonality values (from Table 9)	Largest difference (1-commonality value)	Largest weight	Weights obtained by proportionality
Hardness	0.972	(1–0.972) = 0.028	0.28	0.28
Total Residue	0.962			0.27
Sulphate	0.579			0.17
Fluoride	0.511			0.15
Iron	0.444			0.13
Sum of weights				1.00

Using the communality values, and the procedure defined in this work, the relative weight (wi) for each parameter is: hardness (0.28), total residue (0.27), sulphate (0.17), fluoride (0.15), and iron (0.13). The sum of the five weights add to one (1.00).

Thus, the multivariable analysis helped to define the five parameters to represent the groundwater quality of the state of Bahia; and the weight of importance for each parameter (w_i_), independent of subjective assessments. The next step is to transform the chemical concentration (c_i_) for each variable, in dimensionless grade (q_i_), to calculate the GWQI value for each water sample.

### Nonlinear fit to transform dimensional groundwater quality parameters in dimensionless subindices

**Figure 7:**
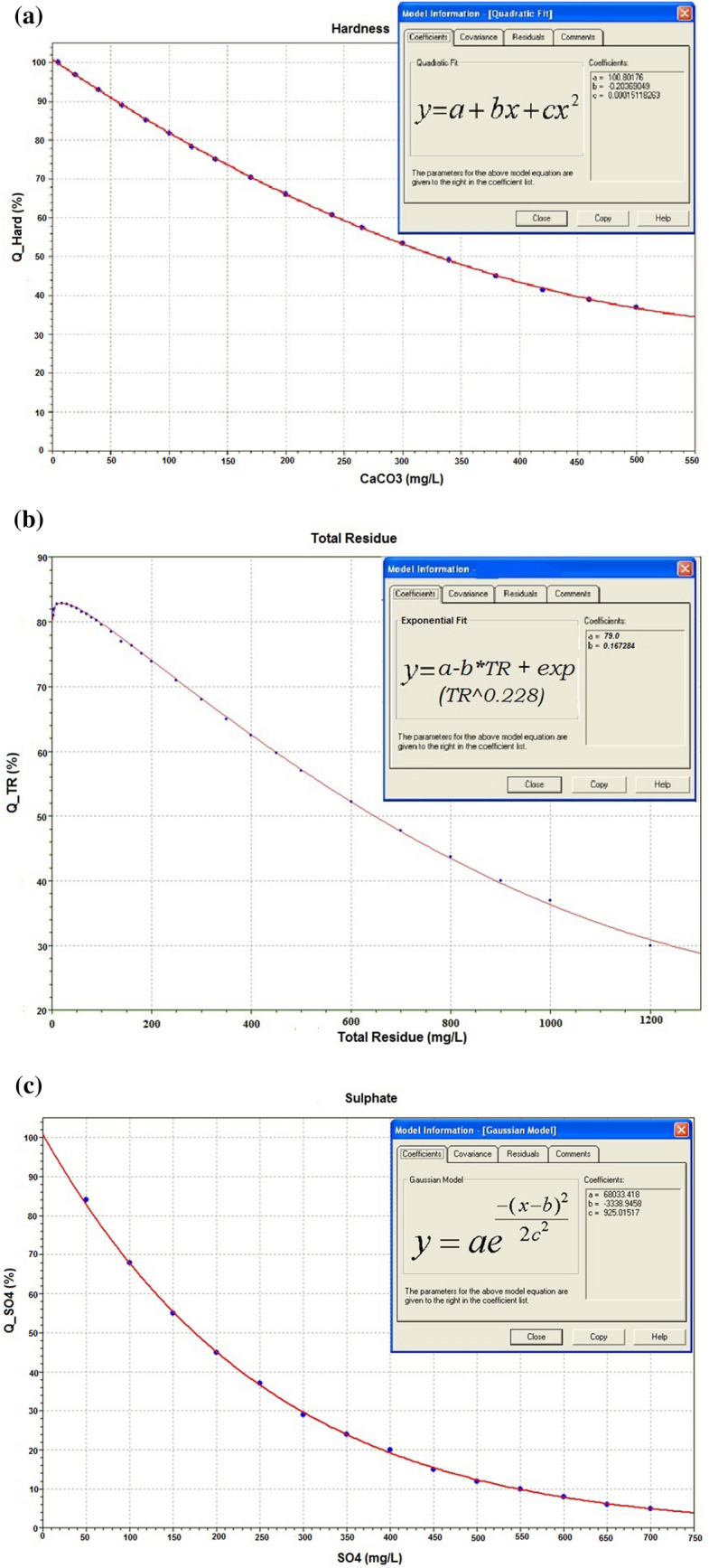

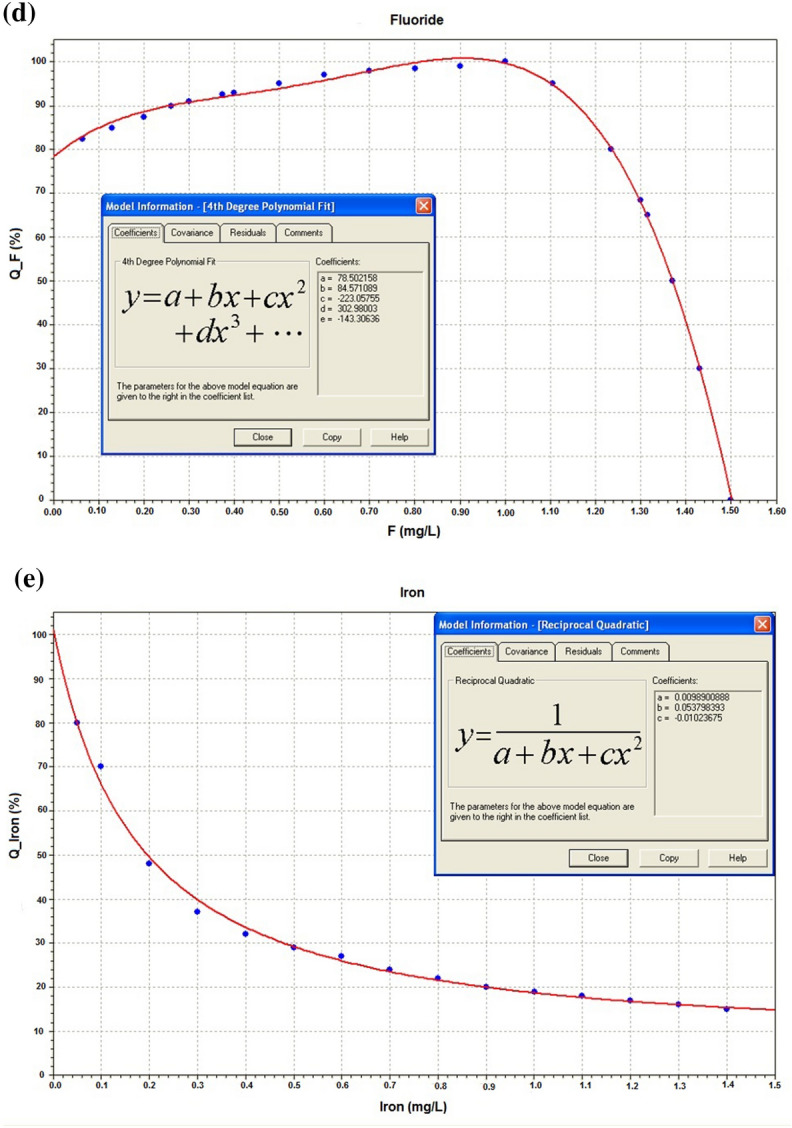
Nonlinear fitting models for the parameters: (**a**) hardness; (**b**) total residue; (**c**) sulphate; (**d**) fluoride; (**e**) iron; using Statgraphics, Centurion XVI^119^.

It was necessary to develop empirical curves, with chemical concentrations in the abscissa and grades (from 0.0 to 100.0) in the ordinate. The grades were defined using the limits for drinking water, from the Resolution 2914/2011^87^. The Fig. 7a–e show the curves (concentration versus grade) for the parameters (hardness, total residue, sulphate, fluoride, and iron), and the mathematical models derived using the nonlinear curve fitting from the statistical package Statgraphics Centurion XVI^119^.

The Table 8 presents the nonlinear fit for the five parameters (hardness, total residue, sulphate, fluoride, and iron), the respective fitting constants, the validity intervals, and the respective correlation coefficients R^2^.

**Table 8:** Nonlinear fitting models for each parameter.

Parameter	Mathematical models	Fitting constant values	Validity intervals	R^2^ (%)
Hardness	$y = a + b x + c x^{2}$	a = 100.8018 b = − 0.2037 c = 0.0002	[5.4 ≤ Hard ≤ 500] If: Hard < 5.4 ⇒ Q_Hard_ = 100 If: Hard > 500 ⇒ Q_Hard_ = 2.6	99.99
Total Residue	$y = a - b x + e^{{x^{0.228} }}$	a = 79.00 b = 0.167	[0 ≤ TR ≤ 1000] If: TR > 1000 ⇒ Q_TR_ = 2.27	99.95
Sulphate	$y = a e^{{\frac{{ - \left( {x - b} \right)^{2} }}{{2c^{2} }}}}$	a = 68,033.42 b = − 3338.95 c = 925.02	[0 ≤ SO_4_^2−^ ≤ 250] If: SO_4_^2−^ > 250 ⇒ Q_SO4_^2−^ = 0.25	99.97
Fluoride	$y = a + bx + cx^{2} + dx^{3} + ex^{4}$	a = 78.50 b = 84.57 c = − 223.06 d = 302.98 e = − 143.31	[0 ≤ F ≤ 1.5] If: F > 1.5 ⇒ Q_F_ = 0.11	99.88
Iron	$y = \frac{1}{{\left( {a + bx + cx^{2} } \right)}}$	a = 0.0099 b = 0.0538 c = − 0.0102	[0 ≤ Iron ≤ 0.30] If: Iron > 0.30 ⇒ Q_Iron_ = 0.04	99.67

### Mathematical formulation for the groundwater quality index

The mathematical formulation for the GWQI is similar to the formulation of the WQI-NSF, a product of grades (q_i_) raised to a power (w_i_), or the degree of importance of each parameter in the water quality (Eq. 1).

1 $${\text{GWQI}} = {\Pi Q}_{{\text{i}}}^{wi} = {\text{Q}}_{{{\text{HARD}}}}^{0.28} \times {\text{Q}}_{{{\text{TR}}}}^{0.27} \times {\text{Q}}_{{{\text{SO}}4}}^{0.17} \times {\text{Q}}_{{\text{F}}}^{0.15} \times {\text{Q}}_{{{\text{IRON}}}}^{0.13}$$

The grades representing the groundwater quality vary from 0.0 to 100.0. The classification of the groundwater quality, based on the GWQI values, are similar to the classification for the WQI-NSF, as follows: grades 0.00–19.99 (BAD, color RED); 20.00–36.99 (POOR, color PINK); 37.00–51.99 (REGULAR, color YELLOW); 52.00–79.99 (GOOD, color GREEN); and, 80.00–100.00 (GREAT, color BLUE).

### Application of the groundwater quality index for the state of Bahia

From the spreadsheet for 1369 wells presented as Supplementary Material, the number of wells with GWQI classified as (BAD, POOR, REGULAR, GOOD and GREAT) for the hydrogeological domains (sedimentary, crystalline, karstic, and metasedimentary) have the following distribution: the grades (BAD and POOR) corresponds to 69.5% of the total number of wells, and (GOOD and GREAT) to 30.1%. The percentage of 69.5% has good correlation with the percentage of wells drilled in the domains (crystalline and karstic) which is 60.3%. These domains are mainly in the arid and semiarid regions of the state of Bahia and produce groundwater of inferior quality.

To investigate if the GWQI values for the sample of 1369 wells (Supplementary Material), are affected by the characteristics of the sample of 600 wells (Supplementary Material), used to develop the GWQI, it was calculated for this sample, the number of wells, per hydrogeological domain, in which, the concentrations for the parameters (hardness, total residue, sulphate, fluoride and iron), are, below and above, the limits for drinking water^87^. The calculations presented in the spreadsheet (600 wells), indicated an averaged percentage of 70.5% for the set (concentrations below the limits); and, averaged percentage of 29.5% for the set (concentrations above the limits). Based on these results, it is expected for the sample of 600 wells, around 70.5% of grades (GOOD and GREAT), and around 29.5% of grades (BAD and POOR). These results are quite different from the sample (1369 wells), with 69.5% (BAD and POOR), and, 30.1% (GOOD and GREAT). The difference between the samples indicates that the calculation of the GWQI, for the 1369 wells, was not biased, and the multivariate process not flawed.

To visualize how the GWQI values, and the respective grades, are correlated with the characteristic of the groundwater sample, Table 9 shows, for ten wells located in the crystalline and karstic hydrogeological domains, the GWQI values and grades, and the concentration for the parameters (hardness, total residue, sulphate, fluoride and iron). The data were taken from the set of 1369 wells (Supplementary Material).

**Table 9:** Ten values of the GWQI calculations with the Eq. (1) and grades from the GWQI and the NGWQI previously derived.

Well Number	Hardness	Total residue	Sulphate	Fluoride	Iron	GWQI value	GWQI grade	NQWQI grade (previous index)
1975	1213.51	2490.0	103.1	0.09	6.42	4.27	BAD	REGULAR
7455	1858.0	4692.0	364.0	0.85	0.05	4.54	BAD	POOR
3012	899.65	922.0	252.5	1.15	0.08	9.53	BAD	GOOD
3162	709.42	1378.0	185.0	0.95	0.05	11.1	BAD	REGULAR
2736	454.72	1326.0	75.0	0.46	0.02	25.82	POOR	REGULAR
5504	340.26	990.0	80.0	1.34	0.22	49.79	REGULAR	GOOD
2153	459.0	836.0	96.6	0.37	0.07	54.31	GOOD	REGULAR
1044	196.0	284.0	15.4	0.23	0.02	77.8	GOOD	GOOD
697	18.32	86.0	2.20	0.14	0.08	87.52	GREAT	GOOD

Drinking water standards (Brazil 2011): hardness = 500 mg/L; total residue = 1000 mg/L; sulphate = 250 mg/L; fluoride = 1.5 mg/L; iron = 0.3 mg/L).

The data on Table 9 show GWQI values from 4.27 to 87.52, very well correlated with the parameters concentration: (i) if parameters have concentrations above the limits, the grades are (BAD and POOR); (ii) if concentrations are close to the limits (REGULAR); and, (iii) if concentrations are below the limits (GOOD and GREAT).

Finally, with the objective to compare the groundwater quality evaluation resulting from the new index (GWQI), with the previous index NGWQI (Oliveira et al. 2007)^71^, it was examined the number of similar and dissimilar results using both indices. These results are presented in the spreadsheet for 1369 wells (Supplementary Material).

Examining the similarity between the grades it was found that: the grades (GOOD and GREAT by NGWQI), have similarities (44.5 and 53.7%) with the grades (GOOD and GREAT by GWQI), which means, around half of the wells had similar groundwater quality evaluation by the two indexes. Significant correspondence was verified only for the inferior grades, for instance, 100% correspondence occurs between (POOR by NGWQI) with (BAD + POOR by GWQI); and 94.2% correspondence occurs between (REGULAR by NGWQI) with (BAD + POOR by GWQI).

The explanation for these results, is that, GWQI and NGWQI have, in common, only the parameters (hardness, total residue, and fluoride). Using the multivariable techniques, the parameters (sulphate and iron) were included in the GWQI, while the parameters (chloride, nitrate, and pH) were discharged. The parameter chloride, though with significant factor load, belongs to the same hierarchical group as hardness; pH has no significant factor load; and nitrate, significant only in (Factor 6), it is not a regionalized variable.

The superiority of GWQI lies in the analytical methodology used for its development, instead subjective assessment, based on experts’ opinion. The multivariable analysis allowed, unequivocally, to include in the index, the most significant parameters to qualify the groundwater of the state of Bahia, besides to indicate the degree of importance, or weight, for each parameter.

**Figure 8:**
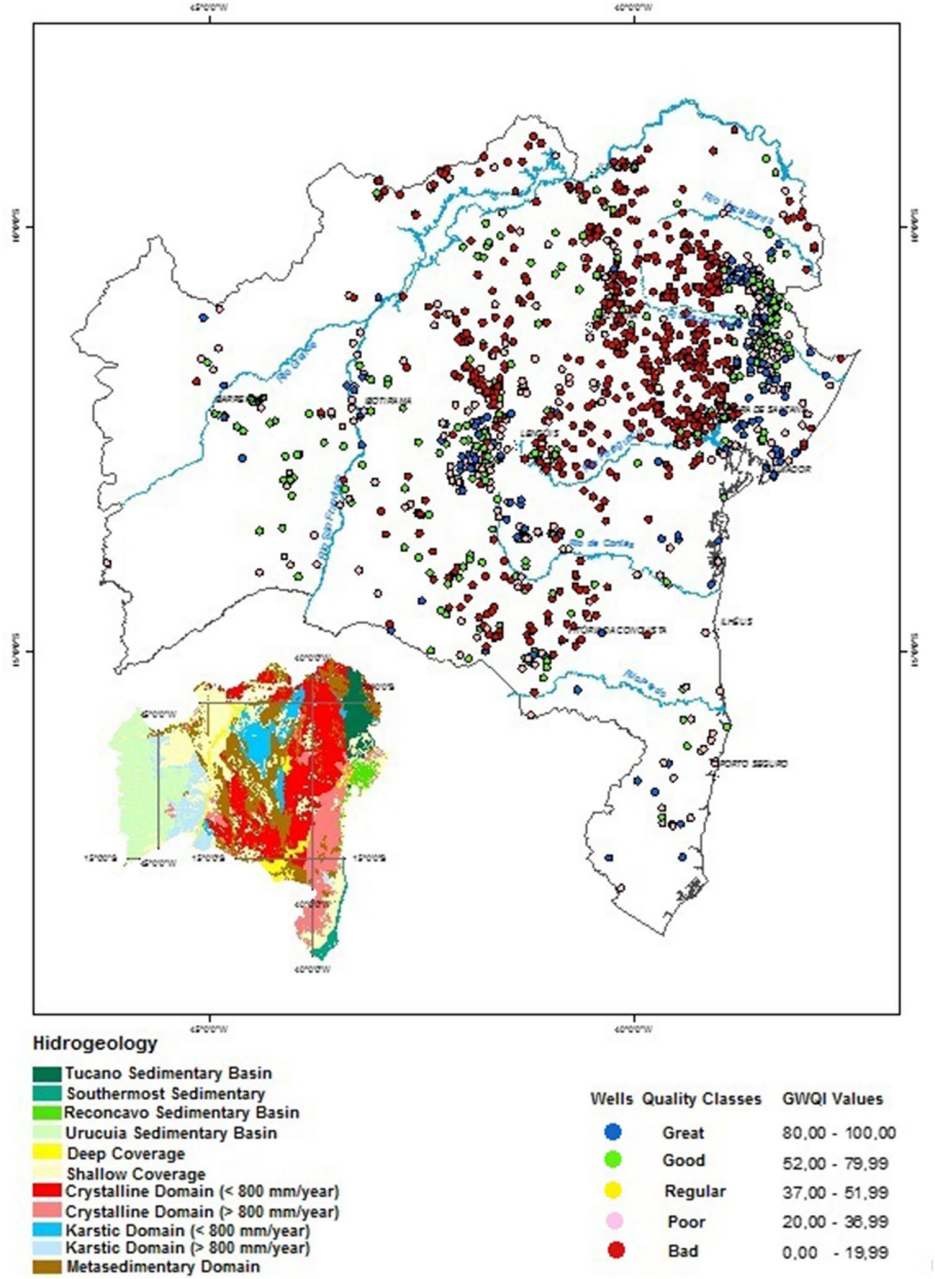
Spatialization of 1369 wells with the respective GWQI color (quality indicator) on the map of the state of Bahia, using ArcView version 9.3^120^. The small view: state of Bahia hydrogeological map (Fig. 1).

The Fig. 8 shows the spatialization of colored dots, on top of the map of the state of Bahia, corresponding to the GWQI grades for the set of 1369 wells.

The Table 10 summarizes the relation between the GWQI colors (quality indicators), the characteristics of the hydrogeologic domains and the groundwater quality, associated to the map of Fig. 8.

**Table 10:** GWQI colors (quality indicators), the hydrogeologic domain, and the respective groundwater quality.

Predominant GWQI color	Predominant GWQI quality	Hydrogeologic domain and colors from map	Water quality and averaged total residue
BLUE and GREEN (with dots in pink & red)	GREAT and GOOD (some poor & bad)	State eastern sedimentary domain: Tucano (very dark green);	Tucano: variable groundwater favorability, the Marizal formation has tendency of salinization in depth Averaged TR (285–725 mg/l)
BLUE and GREEN (with dots in pink & red)	GREAT and GOOD (some poor & bad)	State eastern sedimentary domain: Reconcavo (green);	Reconcavo: store water of good quality but Sergi formation has tendency of salinization in depth Averaged TR (181–285 mg/l)
GREEN (with dots in blue & pink)	GOOD (some great & poor)	State eastern sedimentary domain Southernmost (dark green);	Southernmost: can store water of good quality in the sandstones Averaged TR (182 mg/l)
GREEN (with dots in blue & pink & red)	GOOD (some great & poor & bad)	Western sedimentary (light green)	Western sedimentary: high potentiality and excellent groundwater quality Averaged TR (164 mg/l)
GREEN (with dots in blue & pink & red)	GOOD (some great & poor & bad)	Metasedimentary (in brown);	Metasedimentary: groundwater has from good to inferior quality Averaged TR (1016 mg/l)
GREEN and PINK (with dots in blue & red)	GOOD and POOR (some great & bad)	Karstic with rainfall > 800 mm/year (in light blue)	Karstic: groundwater has from good to inferior quality Averaged TR (661 mg/l)
PINK and GREEN (with dots in blue & red)	POOR and GOOD (some great & bad)	Crystalline with rainfall > 800 mm/year (in pink)	Crystalline: groundwater has from regular to inferior quality Averaged TR (2633 mg/l)
RED (with dots in pink &green)	BAD (some poor & good)	Crystalline with rainfall < 800 mm/year (in red)	Crystalline: groundwater has generally inferior quality Averaged TR (4550 mg/l)
RED (with dots in pink &green)	BAD (some poor & good)	Karstic with rainfall < 800 mm/year (in blue)	Karstic: groundwater has generally inferior quality Averaged TR (1324 mg/l)

The summary on Table 10 reveal good comparison between the groundwater quality and the water quality classification using the GWQI.

## Conclusions

This work had the objective to develop a groundwater quality index (GWQI) using multivariable analysis techniques. The goal was to improve the performance of a previous index (NGWQI) developed by the research group, using a subjective assessment, through the opinion of experts, represented by hydrogeologists from CERB, the state of Bahia well drilling company.

The major steps of the GWQI development i.e. parameter selection and their respective weights, were totally achieved with the techniques of factorial analysis, principal component analysis, and hierarchical cluster analysis. The PCA helped to define the number of five (5) factors (or variables), which explained 63.91% of the cumulative variance, to participate in the GWQI. The matrix of factorial loads, after the normalized Varimax rotation, indicated the nine (9) water quality parameters to participate of the HCA; and the dendrogram helped to select the five parameters to participate in the GWQI (hardness, total residue, sulphate, fluoride and iron). From the set of communality values, the degree of relevance of each parameter was identified, and, the relative weight (w_i_) for each parameter, was determined. Finally, using nonlinear regression, the normalized curves of concentration versus grades allowed to generate the grade (qi) for each variable concentration. Moreover, the multiplicative formula which operates the dimensionless subindex (q_i_) raised to a power (w_i_), or the weight of importance of each variable, allowed to calculate the values for the GWQI.

Comparison between the groundwater quality evaluations resulting from the new index (GWQI), with the previous index (NGWQI) indicated around half of the wells with grades (GOOD and GREAT by NGWQI) with the same grades (GOOD and GREAT by GWQI), which means, the classifications are not exactly the same using the two indexes. The reason is that, the two indexes have in common, only, the parameters (hardness, total residue, and fluoride). The multivariable techniques included in the GWQI the parameters (sulphate and iron) and removed the parameters (chloride, nitrate, and pH) from the previous NGWQI.

The use of multivariable techniques to develop the GWQI is advantageous, as the multivariable analysis allowed, unequivocally, to select the most significant parameters to represent the groundwater quality, and indicated the degree of importance of each parameter. The new index, GWQI, has the ability to represent the groundwater quality of the state of Bahia, using a single mathematical formulation, with the same five parameters, and raised to unique weight, for each parameter.

## Supplementary Information

Supplementary Information 1.

Supplementary Information 2.

## Data Availability

It was submitted to the journal, as Supplementary Material, the spreadsheet with 600 wells used to develop the multivariable analyses, to define the choice of parameters to participate in the GWQI, and the degree of relevance of each parameter. It was also submitted, the spreadsheet with 1369 wells used to test the formulation for the GWQI in the state of Bahia.
